# Formal home care use by older adults: trajectories and determinants in the Lc65+ cohort

**DOI:** 10.1186/s12913-019-4867-6

**Published:** 2020-01-08

**Authors:** Julien Dupraz, Yves Henchoz, Brigitte Santos-Eggimann

**Affiliations:** Center for Primary Care and Public Health (Unisanté), University of Lausanne, Route de la Corniche 10, 1010 Lausanne, Switzerland

**Keywords:** Home care utilization, Determinants, Trajectories, Longitudinal analysis, Older adults

## Abstract

**Background:**

Given the increasing importance of formal home care services in policies dedicated to elder care, there is major interest in studying individuals’ characteristics determining their utilization. The main objective of this research was to quantify, during a 6-year timeframe, home care use trajectories followed by community-dwelling participants in a cohort study of older adults. The secondary objective was to identify factors associated with home care utilization using Andersen’s *Behavioural Model of Health Services Use*.

**Methods:**

We proceeded to an analysis of data prospectively collected in the setting of the Lc65+ population-based study conducted in Lausanne (Switzerland). Self-reported utilization of professional home care in 2012 and 2018 was used to define trajectories during this timeframe (i.e. non-users, new users, former users and continuing users). Bivariable analyses were performed to compare new users to non-users regarding the three dimensions of Andersen’s model (predisposing, enabling and need factors) measured at baseline. Then, binomial logistic regression was used in a series of two hierarchical models to adjust for need factors first, before adding predisposing and enabling factors in a second model.

**Results:**

Of 2155 participants aged between 69 and 78 in 2012, 82.8% remained non-users in 2018, whereas 11.2% started to use professional home care. There were 3.3% of continuing users and 2.7% of former users. New users exhibited a higher burden of physical and psychological complaints, chronic health conditions and functional limitations at baseline. After adjusting for these need factors, odds of home care utilization were higher only in participants reporting a difficult financial situation (OR 1.65, 95% CI 1.12–2.45).

**Conclusions:**

In the setting of a Swiss city, incident utilization of formal home care by older adults appeared to be largely determined by need factors. Modifiable factors like personal beliefs and knowledge about home care services did not play a role. After adjusting for need, odds of becoming home care user remained higher in participants reporting a difficult financial situation, suggesting such vulnerability does not hamper access to professional home care in this specific context.

## Background

Fostered by the challenge of population ageing, formal home care services play a growing role in elder care and are of increasing importance in health policies [1, 2]. Hence, there is major interest in studying individuals’ characteristics determining their utilization. This was performed in previous research using Andersen’s *Behavioural Model of Health Services Use*, which postulates that individual, societal and health system characteristics all influence service utilization [3, 4]. The model identifies predisposing, enabling and need factors. Predisposing factors include socio-demographic characteristics and personal beliefs about health services influencing their use, but not directly responsible for it. Enabling factors are mostly related to the financial and organizational accessibility of health services, as well as the impact of social support. Finally, need factors represent the most immediate cause of utilization and can be either perceived by the individual, or evaluated by a third party. Past research examining the application of Andersen’s model to long-term care highlighted the importance of psychosocial factors such as attitudes and knowledge as determinants of service use [5]. However, as pointed out in a systematic review of studies using Andersen’s model, there is a strong tendency to explore factors that have already been included in previous work even if, like age and sex, they are not directly modifiable by policies [6]. On the opposite, evidence regarding dimensions like personal beliefs and knowledge about professional home care is lacking. Moreover, few longitudinal studies addressed the issue of formal home care use and its determinants, and the generalizability of their results is limited by varying designs and definitions [7–10].

Based on these premises, the main objective of the present research was to establish and quantify, during a defined timeframe, the trajectories followed by participants in the Lc65+ cohort regarding professional home care use. The second objective was to better determine the profile of participants with incident utilization of home care, according to Andersen’s model. In a perfectly well-functioning and accessible healthcare system, one could expect that only need factors would account for service use. Therefore, our intention was to identify such factors in the first instance, and to adjust for them when considering other components of Andersen’s model in a second step. Regarding predisposing and enabling factors, intended purpose was to include in analysis modifiable factors and dimensions for which evidence is missing, like personal beliefs and knowledge about home care services.

## Methods

### Study design and population

Launched in 2004 in Switzerland, the Lausanne cohort 65+ (Lc65+) is an ongoing population-based study primarily investigating the manifestations and evolution of frailty in elders, as well as its related outcomes. Detailed information can be found in this reference describing the study protocol and on the Lc65+ website [11, 12]. This work was a secondary analysis of data prospectively collected in the setting of the Lc65+ study, whose participants were recruited in three distinct years (2004, 2009 and 2014). Each year of recruitment, a randomly selected sample was contacted, representing two thirds of the population aged between 65 and 70 registered in the city of Lausanne, Switzerland (140′000 inhabitants). People with severe cognitive decline, living in institution or nearing end of life were excluded. Approximately half the eligible subjects agreed to be enrolled. All three samples were representative of the target population for age and sex. Lc65+ participants are asked to fill a yearly questionnaire and undergo an interview with an examination every 3 years. A follow-up proxy questionnaire is provided when participants are not able to answer themselves. The occurrence of severe cognitive impairment during follow-up is a ground for exclusion when response by proxy is not possible.

The present research focused on the 2012–2018 period, allowing the use of additional data on personal beliefs regarding health services collected in 2012. Therefore, analyses did not include the 2014 sample, whose relatively young age at recruitment (65 to 70 years) was also not particularly relevant with regard to the research question. All community-dwelling individuals who participated in person and provided information on home care utilization in 2012 were eligible. Given the limited scope of the proxy questionnaire, participants who could not answer by themselves in 2012 were not considered for inclusion. We excluded participants for which information on home care use was missing in 2018. Regarding participants eligible in 2012 who were admitted into a nursing home, died or dropped out before 2018, we considered information on home care utilization of the year preceding institutionalization, death or drop-out when available. This implied a minimal follow-up of 2 years, and hence the exclusion of those who were institutionalized, died or dropped out in 2013.

**Fig. 1:**
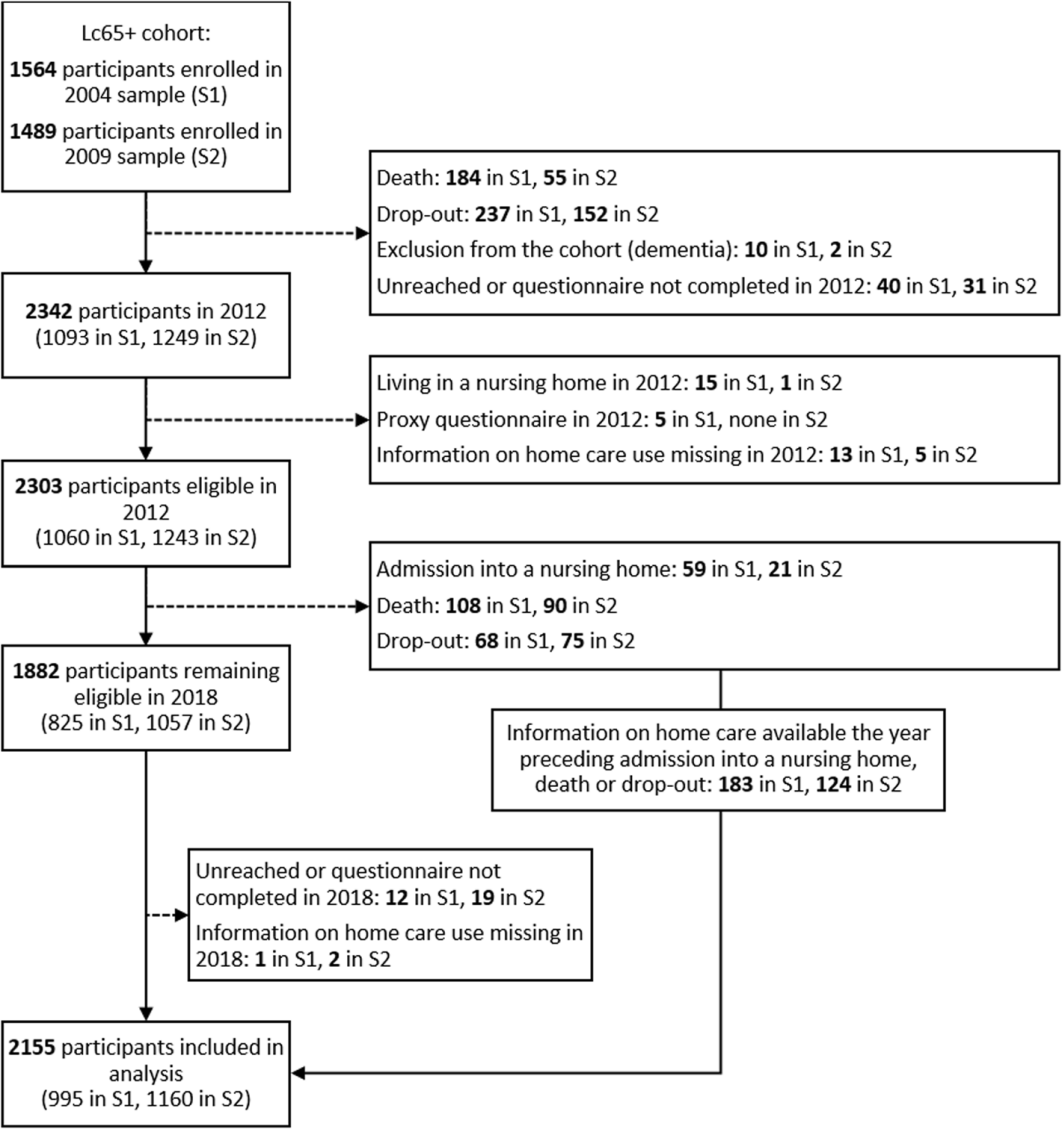
Flow diagram of participation in the study. Legend: S1: 2004 sample (born 1934–1938); S2: 2009 sample (born 1939–1943)

Of the 3053 individuals recruited in the first two samples, 2342 participated in 2012, and 2303 fulfilled eligibility criteria (Fig. 1). During the 2012–2018 period, 80 participants were admitted into a nursing home, 198 died and 143 dropped out. A total of 1882 participants remained eligible in 2018, of which 1848 had information on home care use available and were included. Among those who were institutionalized, died or dropped out between 2012 and 2018, 307 had information on home care utilization of the preceding year available and were included as well. In total, 2155 participants were finally retained in this analysis, namely 93.6% of the 2303 eligible in 2012. We used reporting guidelines developed for observational studies by the STROBE initiative [13].

### Variables

The following question was used to determine self-reported utilization of professional home care, dichotomized as present or absent: “During the last 12 months, have you received, for health reasons, home care or help delivered by professionals?” This information was used to define four trajectories between 2012 and 2018 (or the year before institutionalization, death or drop-out): the non-users in both 2012 and 2018, the non-users in 2012 becoming users in 2018 (new users), the users in 2012 becoming non-users in 2018 (former users), and the users in both 2012 and 2018 (continuing users). Regarding independent variables, the Lc65+ questionnaire was screened for variables considered relevant for the purpose of the present study, based on existing literature and the experience of investigators. Factors belonging to all three dimensions of Andersen’s model were included. Apart from information related to personal history that was collected at enrolment, independent variables were measured by self-report at baseline, in 2012 (unless otherwise specified). The following variables were included (precise formulation of the questions is indicated in the tables):

#### Predisposing factors

i. Demographic: age, sex.
ii. Social: country of birth, educational level.
iii. Beliefs, knowledge and attitudes: general knowledge of professional home care, hesitation to seek home care in case of need, reasons for hesitation.

#### Enabling factors

i. Social support: children, risk for social isolation as measured by the abbreviated version of the Lubben Social Network Scale (LSNS-6; variable assessed in 2011) [14], household composition.
ii. Available income and wealth: difficult financial situation (present when at least one of the following reported: major financial difficulties, healthcare renunciation for economic reasons, entitlement to supplementary benefits to old-age insurance).
iii. Organization and accessibility: length of the relationship with the current attending physician, knowledge of how to seek professional home care.

#### Need factors

i. Perceived: self-rated health, reported physical (pain, dyspnea, urinary incontinence) and cognitive complaints, two-question case-finding instrument for depression (depressed mood and/or anhedonia during the previous month) [15], mobility difficulties (present when reporting difficulty in at least one of the following: walking 100 m, climbing one flight of stairs without stopping, catching something with arms outstretched and higher than shoulders, lifting or carrying more than 5 kg), falls, fear of falling.
  To maximize the adjustment for need in the multivariable analysis, difficulties in activities of daily living (dressing, taking a bath or a shower, feeding, transferring, using the toilet) and instrumental activities of daily living (housekeeping, preparing food, shopping, using the telephone, managing medication, handling money) were assessed both in 2012 and 2018 (or the year before institutionalization, death or drop-out). A categorical variable was created on this basis (no difficulty in 2018, difficulty in one or more ADL/IADL in 2018 but not in 2012, difficulty in one or more ADL/IADL in both 2012 and 2018).
ii. Evaluated: number of diagnosed chronic health conditions (among the following: hypertension, hypercholesterolemia, cardio−/cerebrovascular disease, diabetes, pulmonary disease, osteoporosis, osteoarthritis or other arthritis, malignancy, Parkinson’s disease, Alzheimer’s disease), a selection of five chronic health conditions from this list, unintentional weight loss, frailty phenotype inspired from the Cardiovascular Health Study (based on the following self-reported criteria: weight loss, fatigue, low level of physical activity, difficulty in walking 100 m and/or climbing one flight of stairs without stopping as a proxy for slow walking speed, difficulty in lifting or carrying more than 5 kg as a proxy for weakness; classified as robust if no criterion, pre-frail if 1–2 criteria and frail if 3–5 criteria) [16].

### Statistical analysis

First, we proceeded to a description of baseline characteristics and trajectories of home care use. Statistical significance of differences between 2004 and 2009 samples was evaluated using Kruskal–Wallis test for age and chi-squared test for categorical variables, with a significance threshold of 0.05. Second, we compared the mean (for age) or frequency (for categorical variables) of each independent variable between non-users and new users (similar statistical tests). Both samples were considered together in the bivariable analysis. Third, we conducted several binomial logistic regressions using the same outcome (new users versus non-users), and reported odds ratio with their 95% confidence intervals. Our intention was to adjust for need before adding predisposing and enabling factors in a second step. All need factors exhibiting a *p*-value below 0.2 in the bivariable analysis were included in a first model, and then removed one by one (by decreasing order of *p*-value) until all those remaining had a significant Wald test at the 0.05 threshold. We assessed the non-inferiority of the restricted model compared to the one with all need variables using likelihood-ratio test. Predisposing and enabling factors were then introduced in a second model. Again, only those with a *p*-value below 0.2 in the bivariable analysis were considered. The method of fractional polynomials was used to assess the linearity of age in the logit. In each model, we looked for the presence of interactions between statistically significant variables and used variance inflation factors to detect multicollinearity. Goodness of fit was assessed with the Hosmer-Lemeshow test. Statistical analysis was performed using Stata/IC version 15.1.

### Attrition and sensitivity analysis

Study subjects who dropped out between their recruitment in Lc65+ and 2012 were compared to those participating in 2012 for various socio-demographic characteristics (sex, country of birth, educational level, household composition) and self-rated health at enrolment. Characteristics differing between leavers and participants in 2012 (based on chi-squared test with a significance threshold of 0.05) were used to build strata. The probability of participation in 2012 was estimated in each stratum and used to assign weights to study participants. All analyses were then repeated with inverse probability weighting.

## Results

### Study population

Mean age of the participants, who were between 69 and 78 in 2012, was 73.2 years (76.0 in 2004 sample, 70.8 in 2009 sample) and 60.5% were female (Table 1). More than three quarters (79.5%) knew what a home care organization is and offers, and 57.6% would not have hesitated at all to contact such an organization in case of need. The fear of losing autonomy and privacy was more frequently reported by those hesitating than administrative and financial concerns. Overall, 24.2% were at risk for social isolation (i.e. had a LSNS-6 score below 12) and 41.7% lived alone. Nearly a quarter (24.6%) reported a difficult financial situation. Finally, 62.0% of participants knew how to get professional home care in case of need.

**Table 1:** Trajectories and characteristics of study participants, by sample

	2004 sample (*N* = 995)	2009 sample (*N* = 1160)	Both (*N* = 2155)	N	
Formal home care use trajectory between 2012 and 2018^a^, n (%)				2155	#
Non-users	790 (79.4)	995 (85.8)	1785 (82.8)		
New users	125 (12.6)	116 (10.0)	241 (11.2)		
Former users	36 (3.6)	23 (2.0)	59 (2.7)		
Continuing users	44 (4.4)	26 (2.2)	70 (3.3)		
**Predisposing factors**					
Demographic					
Age in 2012, mean (SD)	76.0 (1.4)	70.8 (1.4)	73.2 (2.9)	2155	#
Female sex, n (%)	600 (60.3)	704 (60.7)	1304 (60.5)	2155	
Social					
Born abroad (vs born in Switzerland), n (%)	239 (24.0)	313 (27.1)	552 (25.7)	2152	
Highest level of educational attainment, n (%)				2152	#
Compulsory education	219 (22.1)	197 (17.0)	416 (19.3)		
Apprenticeship	402 (40.5)	461 (39.7)	863 (40.1)		
High school, university or equivalent	371 (37.4)	502 (43.3)	873 (40.6)		
Beliefs, knowledge and attitudes					
Knows very well or rather well what a home care organization is and what it can offer (vs rather no or not at all), n (%)	691 (80.8)	828 (78.5)	1519 (79.5)	1910	
Would not hesitate at all to seek help from a home care organization in case of need (vs a little, a lot or would not seek help), n (%)	489 (58.3)	595 (57.1)	1084 (57.6)	1881	
Among respondents that would hesitate to seek help from a home care organization (*N* = 797), n (%) afraid of					
- Losing their ability to decide for the organization of everyday life	297 (88.9)	385 (91.2)	682 (90.2)	756	
- Losing their privacy	266 (80.1)	348 (82.9)	614 (81.7)	752	
- Losing their ability to choose who comes home	270 (82.1)	325 (77.9)	595 (79.8)	746	
- Seeing their household “invaded” by several people	274 (82.5)	319 (76.9)	593 (79.4)	747	
- The heaviness of administrative procedures	261 (79.6)	321 (77.0)	582 (78.1)	745	
- The waiting time before receiving help and care	253 (76.7)	307 (73.8)	560 (75.1)	746	
- Looking fragile, impaired	244 (74.9)	304 (72.9)	548 (73.8)	743	
- Home care fees	230 (69.9)	297 (70.9)	527 (70.5)	748	
**Enabling factors**					
Social support					
Without children, n (%)	214 (21.6)	242 (21.0)	456 (21.3)	2143	
Lubben Social Network Scale (LSNS-6) score of less than 12 (vs 12 or more), n (%)	247 (26.0)	231 (22.6)	478 (24.2)	1974	
Lives alone, n (%)	409 (41.4)	485 (42.0)	894 (41.7)	2145	
Available income and wealth					
Difficult financial situation, n (%)	210 (24.8)	250 (24.4)	460 (24.6)	1872	
Organization and accessibility					
Number of years with the current attending physician, n (%)				1868	#
2 years or less	96 (11.3)	138 (13.5)	234 (12.5)		
3 to 10 years	204 (24.1)	293 (28.7)	497 (26.6)		
More than 10 years	548 (64.6)	589 (57.8)	1137 (60.9)		
Knows very well or rather well how to find a home care organization in case of need (vs rather no or not at all), n (%)	550 (64.0)	629 (60.4)	1179 (62.0)	1902	
**Need factors**					
Perceived					
Self-rated health reported as fair, poor or very poor (vs very good or good), n (%)	341 (34.3)	312 (26.9)	653 (30.3)	2155	#
Suffers since at least 6 months from, n (%)				2131	
- Pain in articulations of legs, shoulders, arms or hands, or back pain	632 (64.2)	660 (57.6)	1292 (60.6)		#
- Shortness of breath, difficult breathing	155 (15.7)	139 (12.1)	294 (13.8)		#
- Memory lapses affecting everyday life, difficulty concentrating, or difficulty taking decisions in everyday life	155 (15.7)	155 (13.5)	310 (14.6)		
- Involuntary leakage of urine	125 (12.7)	146 (12.7)	271 (12.7)		
Depressed mood, anhedonia or both during the past 4 weeks, n (%)	259 (26.3)	258 (22.4)	517 (24.2)	2138	#
Mobility difficulties, n (%)	483 (49.0)	464 (40.4)	947 (44.4)	2133	#
Falls not associated with sport activity during the past 12 months, n (%)				2142	#
No	763 (77.2)	947 (82.1)	1710 (79.8)		
Yes, once	174 (17.6)	157 (13.6)	331 (15.5)		
Yes, several times	51 (5.2)	50 (4.3)	101 (4.7)		
Fear of falling, n (%)				2113	#
Not at all	455 (46.7)	641 (56.3)	1096 (51.9)		
Yes, without activity restriction	387 (39.7)	394 (34.6)	781 (37.0)		
Yes, with restriction from certain activities because of it	133 (13.6)	103 (9.1)	236 (11.2)		
Difficulty or need for help in one or more ADL/IADL, n (%)				2065	#
No difficulty or need for help in 2018^a^	506 (53.2)	755 (67.8)	1261 (61.1)		
Difficulty or need for help in 2018^a^, but not in 2012	227 (23.9)	197 (17.7)	424 (20.5)		
Difficulty or need for help in 2012 and 2018^a^	218 (22.9)	162 (14.5)	380 (18.4)		
Evaluated					
Number of diagnosed chronic health conditions^b^ reported during the past 12 months, n (%)				2135	#
0	267 (27.2)	365 (31.7)	632 (29.6)		
1	289 (29.4)	381 (33.1)	670 (31.4)		
More than 1	427 (43.4)	406 (35.2)	833 (39.0)		
Treated for or suffering from the following diagnosed chronic health conditions during the past 12 months, n (%)				2135	
- Coronary artery disease, heart failure, heart valve/muscle disease, or cerebrovascular disease	114 (11.6)	92 (8.0)	206 (9.7)		#
- Diabetes	101 (10.3)	117 (10.2)	218 (10.2)		
- Chronic obstructive pulmonary disease or asthma	89 (9.1)	105 (9.1)	194 (9.1)		
- Osteoarthritis or other types of arthritis	294 (29.9)	282 (24.5)	576 (27.0)		#
- Cancer, malignancy, lymphoma	43 (4.4)	57 (5.0)	100 (4.7)		
Unintentional weight loss during the past 12 months, n (%)	116 (11.8)	120 (10.4)	236 (11.0)	2140	
Frailty phenotype, n (%)				2098	#
Robust	466 (47.9)	608 (54.0)	1074 (51.2)		
Pre-frail	409 (42.1)	460 (40.9)	869 (41.4)		
Frail	97 (10.0)	58 (5.2)	155 (7.4)		

^a^For those who were admitted into a nursing home, died or dropped out before 2018, information of the year preceding institutionalization, death or drop-out was considered

^b^Among the following: hypertension, hypercholesterolemia, cardio−/cerebrovascular disease, diabetes, pulmonary disease, osteoporosis, osteoarthritis or other arthritis, malignancy, Parkinson’s disease, Alzheimer’s disease

^#^Difference between both samples statistically significant at the 0.05 threshold

Regarding predisposing and enabling factors, discrepancies between both samples were subtle, besides the age difference related to the study design. Participants in 2009 sample tended to have a higher educational level and a shorter relationship with their current attending physician. Most need factors were more frequently mentioned by participants in 2004 sample, who had poorer self-rated health and more difficulties in mobility and ADL/IADL, disclosed more falls and chronic health conditions, and were frailer.

### Trajectories of formal home care use: frequencies and bivariable analysis

During the study period, most participants (82.8%) remained non-users, whereas one in ten (11.2%) started to use professional home care, and about 3% were former users (2.7%) or continuing users (3.3%). Overall, 20.6% of participants in 2004 sample mentioned home care use at some point, a significantly higher figure than that observed in 2009 sample (14.2%). Compared with non-users, those becoming users of professional home care were older (73.7 versus 73.1 years old, Table 2), had a higher risk of social isolation according to LSNS-6 (32.9% versus 22.0%), lived more often alone (46.9% versus 39.3%) and were twice more prone to mention a difficult financial situation (39.5% versus 21.1%). We did not find any association with sex, country of birth, educational level, children and the length of the relationship with the general practitioner. Factors directly related to professional home care, be they about individual perception or accessibility, did not show any significant association either. Finally, with the exception of malignancies, all need factors were more commonly reported at baseline by participants becoming users thereafter. This applied to both perceived need (self-rated health, physical and psychological complaints, mobility limitations and falls) and more objective need (diagnosed chronic medical conditions, weight loss and frailty). The correlation with difficulty in one or more ADL/IADL at the end of the study period was obvious (reported by a total of 77.5% of new users, versus 30.8% of non-users).

**Table 2:** Association of predisposing, enabling and need factors with formal home care utilization - bivariable analysis

	Non-users	New users	p	N
	(*N* = 1785)	(*N* = 241)		
**Predisposing factors**				
Demographic				
Age in 2012, mean (SD)	73.1 (2.9)	73.7 (2.9)	0.002	2026
Female sex, n (%)	1069 (59.9)	144 (59.8)	0.968	2026
Social				
Born abroad (vs born in Switzerland), n (%)	448 (25.1)	71 (29.6)	0.138	2023
Highest level of educational attainment, n (%)			0.227	2024
Compulsory education	329 (18.4)	52 (21.7)		
Apprenticeship	711 (39.9)	101 (42.1)		
High school, university or equivalent	744 (41.7)	87 (36.3)		
Beliefs, knowledge and attitudes				
Knows very well or rather well what a home care organization is and what it can offer (vs rather no or not at all), n (%)	1273 (79.7)	153 (74.6)	0.092	1802
Would not hesitate at all to seek help from a home care organization in case of need (vs a little, a lot or would not seek help), n (%)	903 (57.4)	111 (54.7)	0.460	1776
**Enabling factors**				
Social support				
Without children, n (%)	368 (20.7)	54 (22.4)	0.552	2015
Lubben Social Network Scale (LSNS-6) score of less than 12 (vs 12 or more), n (%)	363 (22.0)	69 (32.9)	< 0.001	1859
Lives alone, n (%)	697 (39.3)	113 (46.9)	0.024	2016
Available income and wealth				
Difficult financial situation, n (%)	329 (21.1)	83 (39.5)	< 0.001	1766
Organization and accessibility				
Number of years with the current attending physician, n (%)			0.936	1757
2 years or less	189 (12.2)	24 (11.4)		
3 to 10 years	409 (26.4)	55 (26.2)		
More than 10 years	949 (61.3)	131 (62.4)		
Knows very well or rather well how to find a home care organization in case of need (vs rather no or not at all), n (%)	964 (60.7)	125 (60.1)	0.857	1795
**Need factors**				
Perceived				
Self-rated health reported as fair, poor or very poor (vs very good or good), n (%)	449 (25.2)	121 (50.2)	< 0.001	2026
Suffers since at least 6 months from, n (%)				2002
- Pain in articulations of legs, shoulders, arms or hands, or back pain	1025 (58.1)	166 (69.8)	0.001	
- Shortness of breath, difficult breathing	207 (11.7)	51 (21.4)	< 0.001	
- Memory lapses affecting everyday life, difficulty concentrating, or difficulty taking decisions in everyday life	220 (12.5)	53 (22.3)	< 0.001	
- Involuntary leakage of urine	204 (11.6)	42 (17.7)	0.007	
Depressed mood, anhedonia or both during the past 4 weeks, n (%)	364 (20.5)	90 (38.0)	< 0.001	2010
Mobility difficulties, n (%)	692 (39.2)	154 (64.4)	< 0.001	2005
Falls not associated with sport activity during the past 12 months, n (%)			< 0.001	2014
No	1464 (82.5)	172 (71.7)		
Yes, once	246 (13.9)	48 (20.0)		
Yes, several times	64 (3.6)	20 (8.3)		
Fear of falling, n (%)			< 0.001	1986
Not at all	975 (55.8)	88 (37.1)		
Yes, without activity restriction	643 (36.8)	89 (37.6)		
Yes, with restriction from certain activities because of it	131 (7.5)	60 (25.3)		
Difficulty or need for help in one or more ADL/IADL, n (%)			< 0.001	1940
No difficulty or need for help in 2018^a^	1179 (69.2)	53 (22.5)		
Difficulty or need for help in 2018^a^, but not in 2012	302 (17.7)	98 (41.5)		
Difficulty or need for help in 2012 and 2018^a^	223 (13.1)	85 (36.0)		
Evaluated				
Number of diagnosed chronic health conditions^b^ reported during the past 12 months, n (%)			< 0.001	2008
0	568 (32.1)	46 (19.2)		
1	569 (32.2)	68 (28.3)		
More than 1	631 (35.7)	126 (52.5)		
Treated for or suffering from the following diagnosed chronic health conditions during the past 12 months, n (%)				2008
- Coronary artery disease, heart failure, heart valve/muscle disease, or cerebrovascular disease	141 (8.0)	40 (16.7)	< 0.001	
- Diabetes	152 (8.6)	39 (16.3)	< 0.001	
- Chronic obstructive pulmonary disease or asthma	130 (7.4)	35 (14.6)	< 0.001	
- Osteoarthritis or other types of arthritis	440 (24.9)	81 (33.8)	0.003	
- Cancer, malignancy, lymphoma	81 (4.6)	9 (3.8)	0.559	
Unintentional weight loss during the past 12 months, n (%)	167 (9.4)	37 (15.7)	0.003	2012
Frailty phenotype, n (%)			< 0.001	1971
Robust	977 (56.1)	72 (31.3)		
Pre-frail	687 (39.5)	121 (52.6)		
Frail	77 (4.4)	37 (16.1)		

^a^For those who were admitted into a nursing home, died or dropped out before 2018, information of the year preceding institutionalization, death or drop-out was considered

^b^among the following: hypertension, hypercholesterolemia, cardio−/cerebrovascular disease, diabetes, pulmonary disease, osteoporosis, osteoarthritis or other arthritis, malignancy, Parkinson’s disease, Alzheimer’s disease

### Trajectories of formal home care use: multivariable analysis

In the first model (need factors only), four variables remained significantly associated with the outcome of becoming a home care user (Table 3). Difficulty in one or more ADL/IADL at the end of the study period showed the strongest association, whether new (OR 6.16, 95% CI 4.23–8.98) or already present in 2012 (OR 5.03, 95% CI 3.28–7.72). Pre-frail (OR 1.43, 95% CI 1.01–2.03) and frail participants (OR 2.25, 95% CI 1.29–3.95) had higher odds of becoming users than the robust. Fear of falling at baseline increased the odds of home care utilization only if restriction from certain activities arose from it (OR 1.99, 95% CI 1.25–3.15). Finally, those with cardio−/cerebrovascular disease were more prone to start using home care (OR 1.75, 95% CI 1.13–2.70). There was no significant interaction between variables. The restricted model proved not to be inferior to the one including all need factors (likelihood-ratio test, *p*-value 0.725) and there was no indication of multicollinearity, nor of poor fit (Hosmer-Lemeshow test, *p*-value 0.540).

**Table 3:** Association of predisposing, enabling and need factors with formal home care utilization - logistic regression models

	Odds ratio	95% confidence interval	p
**Model 1: need factors only (*N*** **= 1845)**			
Fear of falling (ref: not at all)			
Yes, without activity restriction	0.97	(0.68–1.37)	0.843
Yes, with restriction from certain activities because of it	1.99	(1.25–3.15)	0.004
Difficulty or need for help in one or more ADL/IADL (ref: none in 2018^a^)			
Difficulty or need for help in 2018^a^, but not in 2012	6.16	(4.23–8.98)	< 0.001
Difficulty or need for help in 2012 and 2018^a^	5.03	(3.28–7.72)	< 0.001
Coronary artery disease, heart failure, heart valve/muscle disease, or cerebrovascular disease	1.75	(1.13–2.70)	0.013
Frailty phenotype (ref: robust)			
Pre-frail	1.43	(1.01–2.03)	0.045
Frail	2.25	(1.29–3.95)	0.005
**Model 2: predisposing, enabling and need factors (*N*** **= 1467)**			
One-year increase in age	1.04	(0.98–1.10)	0.222
Born abroad (ref: born in Switzerland)	1.34	(0.89–2.01)	0.166
Knows very well or rather well what a home care organization is and what it can offer (ref: rather no or not a at all)	1.01	(0.65–1.58)	0.952
Lubben Social Network Scale (LSNS-6) score of less than 12 (ref: 12 or more)	1.24	(0.84–1.85)	0.282
Lives alone (ref: living with other people)	1.16	(0.80–1.69)	0.438
Difficult financial situation	1.65	(1.12–2.45)	0.012
Fear of falling (ref: not at all)			
Yes, without activity restriction	1.19	(0.79–1.80)	0.400
Yes, with restriction from certain activities because of it	2.28	(1.32–3.92)	0.003
Difficulty or need for help in one or more ADL/IADL (ref: none in 2018^a^)			
Difficulty or need for help in 2018^a^, but not in 2012	6.85	(4.44–10.56)	< 0.001
Difficulty or need for help in 2012 and 2018^a^	4.54	(2.69–7.66)	< 0.001
Coronary artery disease, heart failure, heart valve/muscle disease, or cerebrovascular disease	2.06	(1.27–3.37)	0.004
Frailty phenotype (ref: robust)			
Pre-frail	1.26	(0.84–1.89)	0.271
Frail	1.62	(0.80–3.27)	0.178

^a^For those who were admitted into a nursing home, died or dropped out before 2018, information of the year preceding institutionalization, death or drop-out was considered

When adding predisposing and enabling factors in the second model, participants reporting a difficult financial situation at baseline had higher odds of becoming users (OR 1.65, 95% CI 1.12–2.45). Age, country of birth, knowledge of home care organizations, risk of social isolation (i.e. LSNS-6 score below 12) and household composition did not show any association significant at the 0.05 threshold. Need factors retained in model 1 remained significant in model 2, with the exception of the frailty phenotype whose association with the outcome vanished. The relationship between age and the outcome was linear in the logit. Significant variables did not interact. Again, there was no indication of multicollinearity, nor of poor fit (Hosmer-Lemeshow test, *p*-value 0.238).

### Attrition and sensitivity analysis

In both samples, participants were significantly more susceptible to drop out between recruitment and 2012 if they were born abroad, had a lower educational level and mentioned a poorer self-rated health at enrolment. All analyses were repeated with inverse probability weighting based on these three variables, disclosing unchanged results. The only exception was the contribution of the pre-frail state to the first logistic regression model, which was not significant anymore (OR 1.42, 95% CI 0.99–2.03).

## Discussion

In this community-dwelling population in the eighth decade of life, 11.2% started using formal home care over a 6-year timeframe. Compared with non-users (82.8%), they exhibited a higher burden of physical and psychological complaints, chronic health conditions and functional limitations at baseline. After adjusting for these need factors, participants reporting a difficult financial situation had higher odds of incident home care utilization.

Our proportion of new users lies between the findings of a German research regarding transition to formal long-term care (7.6% over a 4-year interval) and those of a Dutch work reporting 12.6% of new home care users (3-year interval) [7, 10]. Most participants were familiar with home care organizations in general, but more than a third would not have known concretely how to get help from them in case of need. Moreover, almost half mentioned some reluctance to seek home care services, both by fear of losing autonomy and for practical considerations (procedures, cost). Even if these factors did not seem to play a role in subsequent utilization, they bring to light the unawareness, and sometimes the mistrust, of a substantial part of the elders towards professional home care.

When applying Andersen’s model to home care use, the preeminence of need factors was obvious. The association with limitations in ADL/IADL has already been extensively established and, in our case, was further strengthened by taking into account their evolution over the study period [7, 8, 17–23]. In a logistic regression model including this variable, the number of other need factors remaining significantly associated with home care utilization was actually limited. Among them was the restriction in daily activities resulting from a fear of falling, but not fear of falling or falls by themselves. This could be an indicator of a higher degree of functional limitation than that measured by the ADL/IADL variable alone. While the association of frailty with home care use has already been shown in cross-sectional studies, to our knowledge, this is the first evidence of such a relationship in a prospective setting [24–26]. Interestingly, this association was not statistically significant in the second model, denoting a possible confounding effect of enabling and predisposing factors. Regarding cardio−/cerebrovascular disease, our observations corroborate those from other research and suggest the existence of specific needs among people suffering from such disorders [9, 17, 19, 20, 27].

In light of the existing literature, the limited influence of predisposing and enabling factors in our setting is remarkable. Although reported in numerous studies, we did not find any association of sex with the outcome [7–9, 17, 21–23, 28, 29]. This could indicate a variable effect of gender according to the local background. Regarding educational level, previous research was equivocal and our findings support an absence of effect [7, 21, 23, 27, 28, 30–32]. If culturally anchored attitudes are at work in our context, their existence is not supported by an association of incident home care utilization with the country of birth. Not studied until now, the length of the relationship with the general practitioner does not seem to be facilitating.

Even more strikingly, the influence of social support (as measured by the mentioning of children, the risk for social isolation according to LSNS-6, and household composition) appears limited in the bivariable analysis, and disappears when adjusting for need factors. This is in contradiction with previous work [7, 8, 18, 19, 21, 22, 27–31, 33]. We can hypothesize that formal home care does not compensate for the lack of close relatives in our context, but rather carries out tasks that are not accomplished by informal carers anyway. Another explanation may be the existence of a residual confounding effect of need factors in other studies. Furthermore, this could explain why the association with age vanishes in the multivariable analysis, suggesting it is not a determinant by itself but instead an indicator of need, contrary to past research [7–10, 17–23, 27–29, 31, 32].

Finally, literature is equivocal regarding the influence of low income on formal home care utilization, sometimes considering it an enabling factor, sometimes a hindering factor [7, 8, 17, 23, 28, 29, 34]. Our observations support the first hypothesis, since participants reporting a difficult financial situation were more prone to become users, even after adjusting for need factors. In Switzerland, most home care organizations are public and reimbursed by mandatory health coverage. Hence, rather than limiting access to home care, shortage of financial resources could act as an additional factor of vulnerability favouring its use.

### Strengths and limitations

The ability to perform a longitudinal analysis of trajectories over a 6-year timeframe in a large sample of elders is a strength of this study. Furthermore, we investigated dimensions for which evidence was missing, such as personal beliefs and knowledge about home care services and their relationship with subsequent utilization. In addition to the foregoing, the use of a two-step multivariable analysis to maximize adjustment for need factors could have allowed us to estimate more accurately the true association of predisposing and enabling factors with formal home care utilization. Finally, the comparability of the results obtained when considering attrition supports the validity of our observations.

However, several limitations need to be acknowledged. First, we were not able to make a strict distinction between home nursing care and other types of support dispensed by professionals, whose determinants could differ. Second, it is a known fact that the role and the organization of home care services show a large variability from a country to another, and even within countries [2, 35]. This and the predominance of city dwellers in our study population could limit the generalizability of our findings to other contexts. In addition, our study population can be considered as relatively young to investigate the topic of home care utilization, and we cannot exclude that findings would differ in an older population. Lastly, the use of two time points to define trajectories did not allow us to capture the multiple transitions an individual could have experienced during the period under study, and some participants categorized as new users could actually have been only temporary users. Although home care use was assessed at intermediate Lc65+ waves, this was mainly performed by interview for one or the other sample (2014 and 2017 for the first sample, 2013 and 2016 for the second sample). Due to lower participation rates in waves with an interview, analyses were restricted to years 2012 and 2018 in order to limit the proportion of missing data on the outcome (home care use), which prevented the use of alternative analysis methods exploiting data of intermediate time points.

## Conclusions

In the setting of a Swiss city, incident utilization of formal home care by older adults appeared to be largely determined by need factors, which is expected from a well-functioning and accessible healthcare system. Modifiable factors like personal beliefs and knowledge about home care services did not play a role. After adjusting for need, odds of becoming home care user remained higher in participants reporting a difficult financial situation, suggesting such vulnerability does not hamper access to professional home care. However, a notable reinforcement of home care offer occurred in the region hosting this study in recent decades, and the applicability of our findings to other political and economic contexts could be limited.

## Data Availability

In order to guarantee the confidentiality of participants, the datasets generated or analysed during the current study are not publicly available. For specific data requests please contact the corresponding author.

## Abbreviations

95% CI: 95% confidence interval
ADL: Activities of daily living
IADL: Instrumental activities of daily living
Lc65+: Lausanne cohort 65+
LSNS-6: Abbreviated version of the Lubben Social Network Scale
OR: Odds ratio

## References

[1] Christensen K, Doblhammer G, Rau R, Vaupel JW (2009). Ageing populations: the challenges ahead. Lancet Lond Engl.

[2] Genet N, Boerma WG, Kringos DS, Bouman A, Francke AL, Fagerström C (2011). Home care in Europe: a systematic literature review. BMC Health Serv Res.

[3] Andersen RM, Newman JF (1973). Societal and individual determinants of medical care utilization in the United States. Milbank Mem Fund Q Health Soc.

[4] Andersen RM, Davidson PL, Baumeister SF, Kominski GF (2014). Improving access to care. Changing the US health care system: key issues in health services policy and management.

[5] Bradley EH, McGraw SA, Curry L, Buckser A, King KL, Kasl SV (2002). Expanding the Andersen model: the role of psychosocial factors in long-term care use. Health Serv Res.

[6] Babitsch B, Gohl D, von Lengerke T (2012). Re-revisiting Andersen’s behavioral model of health services use: a systematic review of studies from 1998–2011. GMS Psychosoc Med.

[7] Steinbeisser K, Grill E, Holle R, Peters A, Seidl H (2018). Determinants for utilization and transitions of long-term care in adults 65+ in Germany: results from the longitudinal KORA-age study. BMC Geriatr.

[8] Slobbe LCJ, Wong A, Verheij RA, van Oers HAM, Polder JJ (2017). Determinants of first-time utilization of long-term care services in the Netherlands: an observational record linkage study. BMC Health Serv Res.

[9] Wong A, Elderkamp-de Groot R, Polder J, van Exel J (2010). Predictors of long-term care utilization by Dutch hospital patients aged 65+. BMC Health Serv Res.

[10] Geerlings SW, Pot AM, Twisk JWR, Deeg DJH (2005). Predicting transitions in the use of informal and professional care by older adults. Ageing Soc.

[11] Santos-Eggimann B, Karmaniola A, Seematter-Bagnoud L, Spagnoli J, Büla C, Cornuz J (2008). The Lausanne cohort Lc65+: a population-based prospective study of the manifestations, determinants and outcomes of frailty. BMC Geriatr.

[12] Lausanne cohorte 65+. https://lc65plus.iumsp.ch/. Accessed 14 July 2019.

[13] von Elm E, Altman DG, Egger M, Pocock SJ, Gøtzsche PC, Vandenbroucke JP (2007). The strengthening the reporting of observational studies in epidemiology (STROBE) statement: guidelines for reporting observational studies. PLoS Med.

[14] Lubben J, Blozik E, Gillmann G, Iliffe S, von Renteln KW, Beck JC (2006). Performance of an abbreviated version of the Lubben social network scale among three European community-dwelling older adult populations. The Gerontologist.

[15] Whooley MA, Avins AL, Miranda J, Browner WS (1997). Case-finding instruments for depression. Two questions are as good as many. J Gen Intern Med.

[16] Fried LP, Tangen CM, Walston J, Newman AB, Hirsch C, Gottdiener J (2001). Frailty in older adults: evidence for a phenotype. J Gerontol A Biol Sci Med Sci.

[17] Li F, Fang X, Gao J, Ding H, Wang C, Xie C (2017). Determinants of formal care use and expenses among in-home elderly in Jing’an district, Shanghai, China. PLoS One.

[18] Murphy CM, Whelan BJ, Normand C (2015). Formal home-care utilisation by older adults in Ireland: evidence from the Irish longitudinal study on ageing (TILDA). Health Soc Care Community.

[19] Wu C-Y, Hu H-Y, Huang N, Fang Y-T, Chou Y-J, Li C-P (2014). Determinants of long-term care services among the elderly: a population-based study in Taiwan. PLoS One.

[20] Borowiak E, Kostka T (2013). Comparative characteristics of the home care nursing services used by community-dwelling older people from urban and rural environments. J Adv Nurs.

[21] de Meijer CAM, Koopmanschap MA, Koolman XHE, van Doorslaer EKA (2009). The role of disability in explaining long-term care utilization. Med Care.

[22] Larsson K, Thorslund M, Kåreholt I (2006). Are public care and services for older people targeted according to need? Applying the Behavioural model on longitudinal data of a Swedish urban older population. Eur J Ageing.

[23] Kemper P (1992). The use of formal and informal home care by the disabled elderly. Health Serv Res.

[24] Hoeck S, François G, Geerts J, Van der Heyden J, Vandewoude M, Van Hal G (2012). Health-care and home-care utilization among frail elderly persons in Belgium. Eur J Pub Health.

[25] Rochat S, Cumming RG, Blyth F, Creasey H, Handelsman D, Le Couteur DG (2010). Frailty and use of health and community services by community-dwelling older men: the Concord health and ageing in men project. Age Ageing.

[26] Vermeiren S, Vella-Azzopardi R, Beckwée D, Habbig A-K, Scafoglieri A, Jansen B (2016). Frailty and the Prediction of Negative Health Outcomes: A Meta-Analysis. J Am Med Dir Assoc.

[27] Portrait F, Lindeboom M, Deeg D (2000). The use of long-term care services by the Dutch elderly. Health Econ.

[28] Plaisier I, Verbeek-Oudijk D, de Klerk M (2017). Developments in home-care use. Policy and changing community-based care use by independent community-dwelling adults in the Netherlands. Health Policy Amst Neth.

[29] Rodríguez M (2014). Use of informal and formal care among community dwelling dependent elderly in Spain. Eur J Pub Health.

[30] Hammar T, Rissanen P, Perälä M-L (2008). Home-care clients’ need for help, and use and costs of services. Eur J Ageing.

[31] Blomgren J, Martikainen P, Martelin T, Koskinen S (2008). Determinants of home-based formal help in community-dwelling older people in Finland. Eur J Ageing.

[32] Stoddart H, Whitley E, Harvey I, Sharp D (2002). What determines the use of home care services by elderly people?. Health Soc Care Community.

[33] Larsson K, Silverstein M (2004). The effects of marital and parental status on informal support and service utilization: a study of older swedes living alone. J Aging Stud.

[34] Wee S-L, Liu C, Goh S-N, Chong WF, Aravindhan A, Chan A (2014). Determinants of use of community-based long-term care services. J Am Geriatr Soc.

[35] Carpenter I, Gambassi G, Topinkova E, Schroll M, Finne-Soveri H, Henrard J-C (2004). Community care in Europe. The aged in home care project (AdHOC). Aging Clin Exp Res.

